# Omega-3 fatty acids, phenolic compounds and antioxidant characteristics of chia oil supplemented margarine

**DOI:** 10.1186/s12944-017-0490-x

**Published:** 2017-05-31

**Authors:** Muhammad Nadeem, Muhammad Imran, Imran Taj, Muhammad Ajmal, Muhammad Junaid

**Affiliations:** 1 Department of Dairy Technology, University of Veterinary and Animal Sciences, Lahore, Punjab 54000 Pakistan; 2 Institute of Home and Food Sciences, Faculty of Science and Technology, Government College University, Faisalabad, Punjab 38000 Pakistan

**Keywords:** Margarine, Omega-3 fatty acids, Chia oil, Phenolic compounds, Sensory evaluation

## Abstract

**Background:**

Chia (*Salvia hispanica* L.) is known as power house of omega fatty acids which has great health benefits. It contains up to 78% linolenic acid (ω-3) and 18% linoleic acid (ω-6), which could be a great source of omega-3 fatty acids for functional foods. Therefore, in this study, margarines were prepared with supplementation of different concentrations of chia oil to enhance omega-3 fatty acids, antioxidant characteristics and oxidative stability of the product.

**Methods:**

Margarines were formulated from non-hydrogenated palm oil, palm kernel and butter. Margarines were supplemented with 5, 10, 15 and 20% chia oil (T_1_, T_2_, T_3_ and T_4_), respectively. Margarine without any addition of chia oil was kept as control. Margarine samples were stored at 5 °C for a period of 90 days. Physico-chemical (fat, moisture, refractive index, melting point, solid fat index, fatty acids profile, total phenolic contents, DPPH free radical scavenging activity, free fatty acids and peroxide value) and sensory characteristics were studied at the interval of 45 days.

**Results:**

The melting point of T_1_, T_2_, T_3_ and T_4_ developed in current investigation were 34.2, 33.8, 33.1 and 32.5 °C, respectively. The solid fat index of control, T_1_, T_2_, T_3_ and T_4_ were 47.21, 22.71, 20.33, 18.12 and 16.58%, respectively. The α-linolenic acid contents in T_1_, T_2_, T_3_ and T_4_ were found 2.92, 5.85, 9.22, 12.29%, respectively. The concentration of eicosanoic acid in T_2_, T_3_ and T_4_ was 1.82, 3.52, 6.43 and 9.81%, respectively. The content of docosahexanoic acid in T_2_, T_3_ and T_4_ was present 1.26, 2.64, 3.49 and 5.19%, respectively. The omega-3 fatty acids were not detected in the control sample. Total phenolic contents of control, T_1_, T_2_, T_3_ and T_4_ samples were 0.27, 2.22, 4.15, 7.23 and 11.42 mg GAE/mL, respectively. DPPH free radical scavenging activity for control, T_1_, T_2_, T_3_ and T_4_ was noted 65.8, 5.37, 17.82, 24.95, 45.42 and 62.8%, respectively. Chlorogenic acid, caffeic acid, quercetin, phenolic glycoside k and phenolic glycoside Q in T_3_ were present 0.78, 0.73, 1.82, 4.12 and 4.49 mg/mL, respectively. After 90 days of storage period, free fatty acids and peroxide value of all the treatments were less than 0.2 (% and MeqO_2_/kg). Sensory characteristics of treatments were not different from the control.

**Conclusion:**

Margarines supplemented with chia oil showed enhanced level of omega-3 fatty acids and antioxidant characteristics. These results suggest that chia oil can be used for formulation of margarine with increased level of omega-3 fatty acids and acceptable sensory characteristics.

## Background

Margarines are characterized on the basis of melting point and hardness [1]. Margarines and shortenings are usually formulated from partially hydrogenated fats which lead to the generation of therapeutically dangerous *t*rans fatty acids. On the basis of scientific information, it has been concluded that *trans* fatty acids do not perform any beneficial biochemical function in the human body [2]. *Trans* fatty acids increase the harmful LDL cholesterol and reduce the beneficial HDL cholesterol. The excessive intake of *trans* fatty acids may lead to cardiovascular diseases, cancer and inflammation [3]. Blending of oils and fats is a straight forward method of developing margarine. It has already been established that physical, chemical characteristics and fatty acid profile of functional margarine product differs from conventional product [4, 5]. On the other hand, with the advancement of knowledge in food nutrition, consumers have started to avoid foods which are formulated from partially hydrogenated fats. Therefore, the demand for functional foods is increasing across the globe [6].

Chia (*Salvia hispanica* L.) has its place from the family *Labiatae* and chia seeds have been the part of human nutrition for about 1500 years BC as staple food in Mexican region [7]. It produces about 35-40% good quality edible oil and possesses the highest concentration of omega-3 fatty acids of all the available food sources [8]. Therefore, it is regarded as powerhouse of omega-3 fatty acids. Scientific evidences have shown that omega-3 fatty acids have cardiac-protective, anti-inflammatory and hypotensive effects [9]. Researchers are trying to improve the functional value of foods by adding omega-3 fatty acids in foods. Consumption of such fortified foods decreased triglycerides level in serum [10]. Numerous efforts have been made to improve the nutritional value of margarine by supplementation with polyunsaturated fatty acids. When improving the nutritional value of margarine, it is extremely important to take into consideration its oxidative acceptability. Polyunsaturated fatty acids are susceptible to auto-oxidation which lead to the generation of characteristics oxidized flavour and toxic oxidation products during the long term storage of fats and oils [11]. During the storage, table margarine usually suffers from the defects of sandiness, surface discoloration, hardening, separation of oil phase and greasy texture [12]. Therefore, the oxidative stability of table margarine is highly important for practical application, nutritional and consumer preferences. Antioxidants are usually added to retard the oxidation in margarine as unrestricted activity of free radicals can lead to atherosclerosis, thrombosis, cancer, accelerated ageing and diabetes [13]. To avoid oxidative stresses, food should be supplemented with polyphenols and beneficial impacts of polyphenols on various biochemical functions of human body have been extensively studied [14]. Earlier investigation has disclosed that chia oil is a good source of phenolic compounds such as cholorogenic acid, caffeic acid, quercetein, phenolic glycoside-K and phenolic glycoside-Q [15]. Azeem et al. [16] studied the impact of chia seed extract for the stabilization of winterized cottonseed oil at ambient temperature. The chia seed extract significantly inhibited the lipid peroxidation and increased the shelf life of cottonseed oil.

Significance of this research work is to develop margarine containing omega-3 fatty acids. Many studies have been performed to improve the fatty acid composition of margarine, however little is known regarding the effect of vegetable oil on linolenic acid, docosahexanoic acid, eicosapentanoic acid, phenolic compounds and antioxidant characteristics of margarine. This study aimed to determine the effect of various concentrations of chia oil on omega-3 fatty acids, phenolic compounds and antioxidant characteristics of table margarine on the basis of chemical and oxidative stability properties.

## Methods

### Materials

Chia seeds were purchased from market of Lahore and oil was extracted from unroasted seeds with laboratory scale expeller. Palm oil, palm kernel oil and palm olein were obtained from United Industries Ltd. Faisalabad. Cultured and unsalted butter was procured from Haleeb Foods, Phool Nagar. Reagents used in this investigation were HPLC grade and purchased from Sigma Aldrich (St. Louis MO, USA).

### Experimental plan

In the present Investigation, T_1_ was comprised of 70% RBD palm oil, 25% non-hydrogenated palm kernel oil and 5% chia oil. T_2_ was comprised of 65% RBD palm oil, 25% non-hydrogenated palm kernel oil, 10% chia oil. T_3_ was comprised of 60% RBD palm oil, 25% non-hydrogenated palm kernel oil and 15% chia oil and T_4_ was comprised of 55% RBD palm oil, 25% non-hydrogenated palm kernel oil and 20% chia oil. Margarine without any addition of chia oil was kept as control. Margarine samples were stored at 5 °C for a period of 90 days. Chemical and sensory characteristics were studied at the interval of 45 days. Experiment was planned in a completely randomized design and each treatment was replicated three times.

### Preparation of margarine

Formulation of margarine was comprised of 82% oil phase, 1% lecithin, 0.6% salt and 16.4% aqueous phase. Ingredients were mixed in respective oil and aqueous phases. Emulsion was formulated by mixing the both phases in electrical blender (Waring blender Model 32BL 80, Dinamic Corporation of America, New Hartford, Connecticut, USA). For the solidification of margarine, emulsion was cooled in 1 l beaker, in ice bath, containing 10% NaCl [17].

### Physical and chemical composition of margarine

Fat, moisture, refractive index, melting point, solid fat index, free fatty acids and peroxide value in margarine samples was determined by following the standard methods [18, 19].

### Fatty acid profile of margarine

Fatty acid methyl esters were prepared by acid transesterification technique using Methanolic HCl (15%) as transesterifying agent. Briefly, 50 mg samples were taken in screw capped test tubes and 2 mL methanolic HCl was added. Tubes were put in a heating block at 100 °C for 60 min and contents of tubes were shaken after every 10 min. After 60 min tubes were cooled to room temperature and 2 mL *n*-hexane and 2 mL deionized water were added. Vortex process was performed at 2200 rpm for exactly 2 min followed by the resting of 15 min and supernatant was dried over anhydrous sodium sulphate. Prepared methyl esters were transferred to GC vials and injected into GC-MS (79890-A Agilent, USA) fitted with fused silica capillary Column (SP 2560; 100 m, film thickness 25 μm) and Mass Selective Detector. Helium was used as carrier gas at the flow rate of 2 mL/min. Fatty acids were identified and quantified by FAME 37 internal standards (Sigma Aldrich, UK) [20].

### Antioxidant characteristics of margarine supplemented with chia oil

#### Total phenolic contents

Total phenolic contents were determined according to the method prescribed by Velioglu et al. [21]. Two hundred and fifty mg samples were mixed with 1.5 mL of 10% solution of Folin-Ciocalteu. After 5 min, 1.5 mL of sodium carbonate (6%) solution were added. Tubes were incubated in the dark for 60 min. Absorbance was read at 760 nm in visible region of spectra on a double bean spectrophotometer (Shimadzu, Japan) against a blank and results were reported as mg gallic acid equivalent per 100 g (mg GAE/g).

#### Scavenger effect on DPPH free radicals

DPPH free radical scavenging activity was determined by following the method of Brand-Williams et al. [22]. 60 mg sample was mixed with 2.44 mL of DPPH solution and samples were incubated at room temperature for 60 min. Absorbance was recorded at 515 nm on a double beam spectrophotometer (Shimadzu, Japan).

#### HPLC characterization of phenolic compounds in margarine

Chlorogenic acid, caffeic acid, quercetin, phenolic glycoside-K and phenolic glycoside-Q were determined on HPLC fitted with quaternary pump and Diode Array Detector. Light sources were deuterium and tungsten lamps on 190-195 nm through reverse phase elution while column specifications were 250 × 4.6 mm, 5 μm (LC-18 column) and 250 mm and 4.6 i.d. (Symmetry C18 column). Mobile phase was comprised of 6% acetic acid prepared in 2 mM sodium acetate and acetonitrile. 10 μL was injected, flow rate was maintained at 1 mL/min and total run time was 75 min. Internal standards of chlorogenic acid, caffeic acid, quercetin, phenolic glycoside-K and phenolic glycoside-Q were prepared in ethanol [23].

#### Oxidative stability during storage

Margarines were stored at 5 °C for 90 days and were sampled at 0, 45 and 90 days. Peroxide value and free fatty acids value was determined according to the standard methods of American Oil Chemists Society [19]. Thriobarbituric acid value was determined at 0, 60, 120 and 180 days of storage period [19] while Induction period was determined at 120 °C with 20 l of oxygen by Professional Rancimat 892 (Metrohm Corporation, Switzerland).

#### Sensory evaluation

Sensory evaluation of margarines supplemented with chia oil was performed by a panel of ten trained judges. Sensory evaluation was performed in well illuminated laboratory at 20 ± 2 °C. Samples of margarines were coded with three digit random number and all the servings were fully randomized. Margarine samples were evaluated for colour, flavour and texture on 9 point scale [24].

### Statistical analysis

The average of the three samples was reported as the measured value with standard deviation. Significant difference among the treatments was determined by Tuckey’s Test. The sample analysis for storage stability and consumer acceptability was carried out in triplicate and the significant differences were calculated among means at a probability level of 5% [25].

## Results and discussion

### Chemical composition, physical and chemical characteristics of margarine supplemented with chia oil

The quality control analysis of substrate oils have been given in Table 1. Fat, moisture and salt content of control and experimental margarines were not different from each other (*p* > 0.05). In our preliminary investigations, chemical composition of control was determined. The ratios of fat, moisture and salt content were adjusted according to the control, which was the reason for non-variation in compositional attributes of margarine. Chemical characteristics of control and different types of margarine are presented in Table 2. Free fatty acids of all the treatments and control were not different from each other (*p* > 0.05). Free fatty acids of RBD palm oil, palm kernel oil, butter and mechanically extracted crude oil from non-roasted chia seeds were 0.10, 0.11, 0.13 and 0.14%, respectively. Lower free fatty acids content of substrate oil led to the lower fatty acids in all the treatments. Free fatty acids are produced as a result of hydrolysis of triglycerides, moisture, lipases, metal ions and temperature. These are considered as the important factors which influence the generation of free fatty acids. Studies of Zhang et al. [26] revealed that free fatty acids of margarine after 12 weeks of storage at 5 °C were less than 0.2% which is the allowable limit for free fatty acids. Supplementation of margarine with chia oil decreased the melting point of all the four treatments (*p* < 0.05). Melting point is an important parameter during the development of table margarine as it helps to determine the spreadibility of margarine after taking out from refrigerator. Melting point provides a temperature indication at which margarine ought to be smooth in the palette. Table (soft) margarine should be immediately spreadable, after taken out from the refrigerator, with no oiling out [27]. International standard range of melting point for the margarine is 28-34 °C which suggests that margarine should quickly melt in the mouth and be firm enough to tolerate the mechanical work during the spreadibility. Melting point of T_1_, T_2_, T_3_ and T_4_ developed in current investigation were 34.2, 33.8, 33.1 and 32.5 °C, which were within the range of international standards. Solid fat index indicates the percentage of triglycerides solidified at a particular temperature. It is an important indicator of many features of foods together with appearance; spreadability and mouth feel of margarine. It also measures the degree of crystallization of fats [28]. Solid fat content is an extremely useful parameter in the formulation of margarine at 10 °C. It determines the hardness of the finished product at refrigeration conditions [29]. Concentration of solid fat in margarine at 10 °C should be more than 10% to prevent oiling off. At 10 °C, amount of solid fat in all the treatments was more than 10%. At 20 °C, solid fat index of control, T_1_, T_2_, T_3_ and T_4_ were 47.21, 22.71, 20.33, 18.12 and 16.58%, respectively (Table 3). At 30 °C, solid fat index of control, T_1_, T_2_, T_3_ and T_4_ were 16.42, 4.52, 3.18, 2.82 and 2.21%, respectively. At 37 °C, Solid fat content of margarine should be less than 6% [30]. In current investigation, solid fat index of all the treatments at 37 °C at was less than 6% which shows that these margarines melt quickly in mouth [17]. Formulated *trans* free margarine from highly saturated soybean oil and evaluation on nuclear magnetic resonance revealed only a small percentage of solid fat above 33 °C. Özay et al. [31] described that solid fat content of soft tub margarine at 20 and 30 °C was 8.5 and 2.5%. Iodine value determines the degree of unsaturation in oils and fats and connected with oxidative stability [32]. Supplementation of margarine with chia oil considerably increased the iodine value of all the treatments. Iodine value of T_4_ was 20.4 points greater than control. Oils having higher iodine values are usually susceptible to auto-oxidation. Peroxide value and refractive index of all the treatments and control were not different from each other (*p* > 0.05).

**Table 1:** Quality control analysis of substrate oils

Parameter	Palm Oil	Palm Kernel Oil	Chia Oil
Free Fatty Acids% (oleic Acid)	0.09 ± 0.01^b^	0.10 ± 0.02^b^	0.14 ± 0.01^a^
Saponification Value (mg KOH/g)	194 ± 1.44^b^	216 ± 4.55^a^	188 ± 2.73^b^
Unsapnifiable Matter%	0.68 ± 0.07^a^	0.65 ± 0.03^a^	1.17 ± 0.09^b^
Peroxide Value (MeqQ_2_/kg)	0.18 ± 0.02^a^	0.21 ± 0.04^a^	0.22 ± 0.05^a^
Iodine Value Cg/100 g (Wijs)	53.2 ± 1.29^b^	18.7 ± 0.59^c^	195.4 ± 1.21^a^

Values represent the mean ± standard deviation; *n* = 3

Means in a row with different superscript letters were significantly different (*p* < 0.05)

**Table 2:** Physical and chemical characteristics of margarine supplemented with chia oil

Parameter	Control	T_1_	T_2_	T_3_	T_4_
FFA %	0.11 ± 0.02^a^	0.11 ± 0.02^a^	0.12 ± 0.01^a^	0.11 ± 0.02^a^	0.12 ± 0.02^a^
MP ^o^C	35.4 ± 0.22^a^	34.0 ± 0.15^b^	33.8 ± 0.25^c^	33.2 ± 0.18^d^	32.1 ± 0.21^e^
IV Cg/100 g	47.5 ± 1.48^e^	57.6 ± 1.21^d^	60.15 ± 1.10^c^	65.6 ± 0.71^b^	70.9 ± 0.52^a^
PV (MeqO_2_/kg)	0.22 ± 0.07^a^	0.25 ± 0.01^a^	0.26 ± 0.05^a^	0.21 ± 0.03^a^	0.24 ± 0.02^a^
RI @ 40 °C	1.454 ± 0.02^a^	1.457 ± 0.01^a^	1.459 ± 0.02^a^	1.463 ± 0.03^a^	1.467 ± 0.02^a^

Values represent the mean ± standard deviation; *n* = 3

Means in a row with different superscript letters were significantly different (*p* < 0.05)

*FFA* Free Fatty Acids (Oleic Acid)

*MP* Melting Point ^o^C

*IV* Iodine Value (Wijs)

*PV* Peroxide Value (MeqO_2_/Kg)

*RI* Refractive Index at 40 °C

Control: Margarine Sample without Chia Oil

T_1_: Margarine Supplemented with 5% Chia Oil

T_2_: Margarine Supplemented with 10% Chia Oil

T_3_: Margarine Supplemented with 15% Chia Oil

T_4_: Margarine Supplemented with 20% Chia Oil

**Table 3:** Solid fat index of margarine supplemented with chia oil

Temperature ^o^C	Control	T_1_	T_2_	T_3_	T_4_
10	60.54 ± 1.27^a^	51.92 ± 1.19^b^	47.46 ± 1.51^c^	44.55 ± 1.41^d^	42.36 ± 1.36^e^
20	47.21 ± 1.23^a^	22.71 ± 1.26^b^	20.33 ± 1.78^b^	18.12 ± 1.21^c^	16.58 ± 1.11^d^
25	39.51 ± 0.88^a^	12.45 ± 0.72^b^	10.15 ± 0.0.45^b^	9.70 ± 0.63^c^	7.44 ± 1.31^d^
30	16.4 ± 0.33^a^	4.52 ± 0.52^b^	3.18 ± 0.0.39^b^	2.82 ± 0.63^c^	2.19 ± 0.28^d^
35	12.3 ± 0.47^a^	3.16 ± 0.63^b^	2.91 ± 0.22^c^	2.51 ± 0.09^c^	2.21 ± 0.11^d^
37	4.52 ± 0.12^a^	2.36 ± 0.10^b^	2.11 ± 0.07^c^	1.84 ± 0.13^d^	1.52 ± 0.13^e^

Values represent the mean ± standard deviation; *n* = 3

Means in a row with different superscript letters were significantly different (*p* < 0.05)

Control: Margarine Sample without Chia Oil

T_1_: Margarine Supplemented with 5% Chia Oil

T_2_: Margarine Supplemented with 10% Chia Oil

T_3_: Margarine Supplemented with 15% Chia Oil

T_4_: Margarine Supplemented with 20% Chia Oil

### Fatty acid profile of margarine supplemented with chia oil

**Fig. 1:**
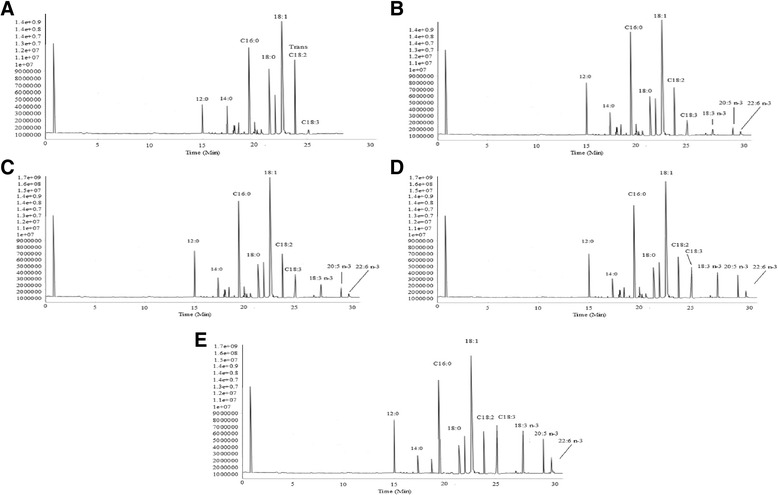
Fatty Acid Profile of Margarine Samples (**a**: Without Chia Oil; **b**: Supplemented with 5% Chia Oil; **c**: Supplemented with 10% Chia Oil; **d**: Supplemented with 15% Chia Oil; **e**: Supplemented with 20% Chia Oil)

Results of fatty acid profile of margarines supplemented with chia oil are given in Table 4 and chromatographs of GC analysis have been presented in Fig. 1. Concentrations of short-chain fatty acids in experimental samples were not different from each other, whereas, they were not detected in control. Concentration of butter in all the experimental samples was 10%, which was the reason for non-significant variation in the concentration of short-chain fatty acids. Role of short-chain fatty acids in the development of typical flavour characteristics of milk and dairy products has been well established. Addition of butter in margarine improved the flavour of margarine. Margarines are usually manufactured from the blends of partially hydrogenated fats and soft oils. Partial hydrogenation of fats and oils lead to the generation of harmful *trans* fatty acids. Partially hydrogenated fats are the major carrier of *trans* fatty acids in human body. Harmful impacts of *trans* fatty acids on serum cholesterol level and cardiovascular diseases have been scientifically proven [3]. Concentration of *trans* fatty acids in control was 18.72% while experimental samples did not reveal trans fatty acids. Nadeem et al. [33] analyzed the concentration of *trans* fatty acids in partially hydrogenated vegetable fat available in Pakistan. The concentration of *trans* fatty acids was greater than 20%. Dollahs et al. [34] improved the concentration of oleic acid in palm stearin and palm kernel oil by *Moringa oleifera* oil through interesterification. The blends thus formulated were free of *trans* fatty acids.

**Table 4:** Fatty acid profile of margarine supplemented with chia oil

Fatty Acid	Control	T_1_	T_2_	T_3_	T_4_
C_12:0_	4.59 ± 0.12^b^	11.25 ± 0.51^a^	10.92 ± 0.19^a^	10.63 ± 0.28^a^	11.17 ± 0.66^a^
C_14:0_	5.42 ± 0.13^a^	5.23 ± 0.08^a^	4.91 ± 0.05^a^	4.58 ± 0.12^a^	4.52 ± 0.06^a^
C_16:0_	11.39 ± 0.29^c^	29.9 ± 1.12^a^	28.87 ± 0.98^a^	27.72 ± 0.55^b^	26.43 ± 0.72^b^
C_18:0_	15.76 ± 0.43^a^	7.27 ± 0.11^b^	7.11 ± 0.19^b^	6.98 ± 0.16^b^	6.55 ± 0.08^b^
C_18:1_	24.88 ± 0.68^d^	34.12 ± 1.73^a^	33.03 ± 1.59^a^	32.42 ± 0.84^b^	30.24 ± 1.35^c^
C_18:2_	18.72 ± 0.54^a^ [*Trans*]	10.28 ± 0.44^b^	10.23 ± 0.37^b^	10.15 ± 0.76^b^	10.12 ± 0.35^b^
α-Linolenic Acid	ND	2.92 ± 0.08^d^	5.85 ± 0.34^c^	9.22 ± 0.24^b^	12.29 ± 0.51^a^
Eicosanoic Acid	ND	1.82 ± 0.05^d^	3.52 ± 0.15^c^	6.43 ± 0.27^b^	9.81 ± 0.32^a^
Docosahexanoic Acid	ND	1.26 ± 0.07^d^	2.64 ± 0.11^c^	3.49 ± 0.13^b^	5.19 ± 0.17^a^

Values represent the mean ± standard deviation; *n* = 3

Means in a row with different superscript letters were significantly different (*p* < 0.05)

*ND* Not detected

Control: Margarine Sample without Chia Oil

T_1_: Margarine Supplemented with 5% Chia Oil

T_2_: Margarine Supplemented with 10% Chia Oil

T_3_: Margarine Supplemented with 15% Chia Oil

T_4_: Margarine Supplemented with 20% Chia Oil

Concentrations of α-linolenic acid in T_1_, T_2_, T_3_ and T_4_ were 2.92, 5.85, 9.22, 12.29%, respectively. Concentration of eicosanoic acid in T_2_, T_3_ and T_4_ were 1.82, 3.52, 6.43 and 9.81%, respectively. While content of docosahexanoic acid in in T_2_, T_3_ and T_4_ were 1.26%, 2.64, 3.49 and 5.19%, respectively. Omega-3 fatty acids were not detected in the control. Health benefits associated with the intake of foods containing omega-3 fatty has led the researchers to formulate the functional containing omega-3 fatty acids. Concentration of omega-3 fatty acids in chia oil and its olein fraction was greater than 60 and 80% [15]. Chia seed has been declared as Novel Food by the European Union Parliament [35]. Scientific studies have evidenced that chia oil is edible with no any toxicological effects and snacks, cereal bars, yoghurt, pasta and biscuits etc. have been supplemented with chia [36]. Chia oil is regarded as the powerhouse of omega-3 fatty acids and the cardio-protective effects of eicosapentanoic acid and docosahexanoic acid have been cited in literature [37]. Concentration of conjugated linoleic acid (Cis-9, *trans*-11; CLA) in T_1_, T_2_, T_3_ and T_4_ were 0.038, 0.035, 0.037 and 0.034%, whereas, CLA was not found in control. Anticarcinogenic, anti-diabetic, immunomodulating, antiatherogenic perspectives of CLA have been published in literature [38]. Daily dose of 3 g of CLA can help to prevent carcinogenesis [39]. Fatty acid composition of margarine has been improved in many studies, however, in current investigation, functional margarine was developed with higher amount of omega-3 fatty acids, CAL and no *trans* fatty acids.

### Storage effect on fatty acid profile

Transition in fatty acid profile of fats and oils during the storage is a good indication of oxidative stability [40]. Concentrations of omega-3 & 6 fatty acids decreased during the storage period of 90 days (Table 5). After 90 days of storage at 5 °C, losses of omeg-6 fatty acids in T_1_, T_2_, T_3_ and T_4_ were 0.04, 0.14, 0.24 and 0.21%, respectively, from the initial values. After 90 days of storage at 5 °C, losses of eicosanoic acid in T_1_, T_2_, T_3_ and T_4_ were 0.07, 0.11, 0.15 and 0.18%, respectively, from the initial values. Chlorogenic acid, caffeic acid, quercetein, phenolic glycoside-k and phenolic glycoside-Q are the major phenolic compounds present in chia oil. Although the concentration of polyunsaturated fatty acids was higher in margarines, however, phenolic compounds of chia oil strongly inhibited the lipid peroxidation in supplemented margarines. Among the food products, margarine is a typical example, which is susceptible to oxidation, because of 82% fat content [30]. Oxidation is a serious problem during the manufacturing and conservation of margarine. The most significant consequence is the development of objectionable odors which usually lead to the lower consumer acceptability [41]. For the prevention of oxidation in margarine, the processing industries normally add vitamin E. Results of current investigation have shown that phenolic substances of chia oil efficiently inhibited the oxidation of polyunsaturated fatty acids and inhibited the development of off-flavors which is also evident from the sensory score of margarine during the storage period. These results suggest that oxidative stability of margarine supplemented with chia oil was remarkable at 5 °C, for a storage period of 90 days. Zhang et al. [26] studied the storage stability of margarine produced from enzymatically tailored fats and orthodox methods. Margarines stored at 5 °C for 12 did not develop oxidative and hydrolytic rancidity. Nadeem et al. [42] monitored the changes in fatty acid profile of palm and mango kernel oil blends for a period of 90 days and the concentration of long-chain unsaturated fatty acids decreased during the storage period (*p* > 0.05). Fatty composition of blends of butter oil and mango kernel oil in ambient and accelerated oxidation (25 and 55 °C) was different from the fresh samples [43].

**Table 5:** Transition in fatty acid profile of margarine supplemented with chia oil

Fatty Acid	Control		T_1_		T_2_		T_3_		T_4_	
	O Day	90* Days	O Day	90 Days	O Day	90 Days	O Day	90 Days*	O Day	90 Days
C_12:0_	4.59 ± 0.12^b^	----	11.25 ± 0.51^a^	10.19 ± 0.32^b^	10.92 ± 0.19^a^	9.48 ± 0.55^e^	10.63 ± 0.28^a^	9.32 ± 0.12^f^	11.17 ± 0.66^a^	8.82 ± 0.43^g^
C_14:0_	5.42 ± 0.13^a^	----	5.23 ± 0.08^a^	4.58 ± 0.02^a^	4.91 ± 0.05^a^	4.55 ± 0.03^a^	4.58 ± 0.12^a^	4.49 ± 0.07^a^	4.52 ± 0.06^a^	4.41 ± 0.06^b^
C_16:0_	11.39 ± 0.29^c^	11.28 ± 0.73^e^	29.9 ± 1.12^a^	29.32 ± 0.79^a^	28.87 ± 0.98^a^	28.42 ± 0.59^b^	27.72 ± 0.55^b^	27.39 ± 1.27^c^	26.43 ± 0.72^b^	26.19 ± 0.64^d^
C_18:0_	15.76 ± 0.43^a^	15.39 ± 1.28^a^	7.27 ± 0.11^b^	6.69 ± 0.05^b^	7.11 ± 0.19^b^	4.57 ± 0.04^b^	6.98 ± 0.16^b^	4.52 ± 0.03^b^	6.55 ± 0.08^b^	4.49 ± 0.08^b^
C_18:1_	24.88 ± 0.68^c^	28.43 ± 1.39^f^	34.12 ± 1.73^a^	33.81 ± 1.16^a^	33.03 ± 1.59^a^	32.58 ± 0.73^b^	32.42 ± 0.84^b^	31.89 ± 0.05^c^	30.24 ± 1.35^c^	28.46 ± 0.89^f^
C_18:2_	18.72 ± 0.54^a^ [*Trans*]	18.13 ± 1.06^a^ [*Trans*]	10.28 ± 0.44^b^	10.13 ± 0.06^b^	10.23 ± 0.37^b^	10.13 ± 0.02^b^	10.15 ± 0.76^b^	9.96 ± 0.35^b^	10.12 ± 0.35^b^	9.76 ± 0.57^b^
α-Linolenic Acid	ND	ND	2.92 ± 0.08^d^	2.88 ± 0.05^d^	5.85 ± 0.16^c^	5.71 ± 0.13^c^	9.22 ± 0.21^b^	8.98 ± 0.31^b^	12.29 ± 0.27^a^	12.08 ± 0.62^a^
Eicosanoic Acid	ND	ND	1.82 ± 0.05^d^	1.75 ± 0.14^d^	3.52 ± 0.15^c^	3.41 ± 0.14^c^	6.43 ± 0.27^b^	6.28 ± 0.22^b^	9.81 ± 0.32^a^	9.63 ± 0.44^a^
Docosahexanoic Acid	ND	ND	1.26 ± 0.07^d^	1.19 ± 0.04^d^	2.64 ± 0.11^c^	2.53 ± 0.16^c^	3.49 ± 0.13^b^	3.35 ± 0.03^b^	5.19 ± 0.17^a^	4.94 ± 0.28^a^

Values represent the mean ± standard deviation; *n* = 3

Means in a row with different superscript letters were significantly different (*p* < 0.05)

*ND* Not detected

Control: Margarine Sample without Chia Oil

T_1_: Margarine Supplemented with 5% Chia Oil

T_2_: Margarine Supplemented with 10% Chia Oil

T_3_: Margarine Supplemented with 15% Chia Oil

T_4_: Margarine Supplemented with 20% Chia Oil

### Antioxidant content of margarine supplemented with chia oil

#### Total phenolic contents of margarine

Total phenolic contents of control, T_1_, T_2_, T_3_ and T_4_ were 0.27, 2.22, 4.15, 7.23 and 11.42 mg GAE/mL, respectively. The higher phenolic contents of experimental margarines are in line with earlier investigations on chia seed. Total phenolic contents of chia seed extract and chia oil were 35 and 7.6 mg GAE/mL, respectively [16]. Total phenolic contents of chia oil and chia seed extract were greater than *Moringa oleifera* oil and sesame cake extract, 7.1 and 1.84%, respectively [33, 44]. Higher total phenolic content in chia oil can be attributed to existence of chlorogenic acid, caffeic acid, quercetin, phenolic glycoside P&K, which were also confirmed by HPLC characterization of phenolic compounds of chia oil. Oxidative stability of margarine can be enhanced by the phenolic compounds of plant origin [45]. Antioxidant characteristics of margarine supplemented with fennel seed extract was superior to the un-supplemented margarine [46]. Supplementation of Shea butter with natural antioxidants improved the antioxidant characteristics [47].

#### DPPH free radical scavenging activity

Determination of DPPH free radical scavenging activity is one of the most effective and widely used methods for the assessment of antioxidant activity of antioxidants of plant origin. In current investigation, DPPH free radical scavenging activity of 100 ppm BHT, control, T_1_, T_2_, T_3_ and T_4_ were 65.8, 5.37, 17.82, 24.95 and 62.80%, respectively. EC_50_ for T_4_ was 1.25 mg/mL as compared to EC_50_ of BHT 1.13 mg/mL. Phenolic substances of chia oil efficiently inhibited the lipid peroxidation, which is also evident form the lower peroxide value while flavour score and peroxide value were strongly correlated (R^2^ = 0.9982). Supplementation of margarine and butter with chokeberry polyphenols extract enhanced the antioxidant activity of fat matrix in cookies and altered the fat oxidation during the storage period of 9 weeks [48]. With a k_2_ value of 37.35 L/moL, vitamin E is regarded as one of the most antioxidant in butter and margarine. Earlier investigation has shown that k_2_ value of Camu-camu (*Myrciaria dubia*) was 69.24 ± 5.72 L/moL, which indicate the possibility of using phytochemicals for the preservation of fats and oils [49].

#### HPLC characterization of phenolic compounds of margarine

**Fig. 2:**
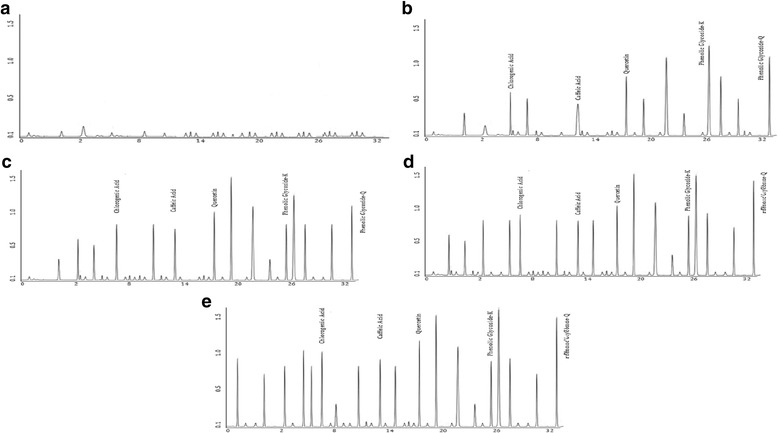
Phenolic Compounds Profile of Margarine Samples (**a**: Without Chia Oil; **b**: Supplemented with 5% Chia Oil; **c**: Supplemented with 10% Chia Oil; **d**: Supplemented with 15% Chia Oil; **e**: Supplemented with 20% Chia Oil)

HPLC characterization of margarines supplemented with chia oil revealed that the chlorogenic acid, caffeic acid, quercetin, phenolic glycoside k and phenolic glycoside Q were the major phenolic compounds (Fig. 2). Concentrations of chlorogenic acid, caffeic acid, quercetin, phenolic glycoside k and phenolic glycoside Q, in T_1_ were 0.33, 0.22, 0.42, 1.07 and 0.62 mg/mL, respectively. Concentrations of chlorogenic acid, caffeic acid, quercetin, phenolic glycoside k and phenolic glycoside Q, in T_2_ were, 0.49, 0.25, 0.69, 1.42 and 1.32 mg/mL. Concentrations of chlorogenic acid, caffeic acid, quercetin, phenolic glycoside k and phenolic glycoside Q, in T_3_ were 0.78, 0.73, 1.82, 4.12 and 4.49 mg/mL. Concentrations of chlorogenic acid, caffeic acid, quercetin, phenolic glycoside k and phenolic glycoside Q, in T_4_ were, 1.21, 0.83, 2.91, 5.23 and 5.58 mg/mL. Whereas, these phenolic compounds were not detected in control samples. During orthodox processing of oils and fats, they are exposed to higher temperature while phenolic compounds are usually lost during the commercial processing of oils and fats. HPLC characterization of ethanolic chia seed extract revealed the existence of chlorogenic acid, caffeic acid, quercetin, phenolic glycoside-k and phenolic glycoside-Q [50]. Health benefits associated with the intake of polyphenols of plant origin have been scientifically established [51]. Earlier investigations regarding the phytochemical characterization of chia oil revealed that it possesses a wide range of phenolic compounds [52]. Chia seed is potentially a strong source of natural antioxidants and phytochemicals of chia can be utilized for the prevention of oxidative stresses in human body and lipid peroxidation [53].

#### Oxidative stability of margarine

**Fig. 3:**
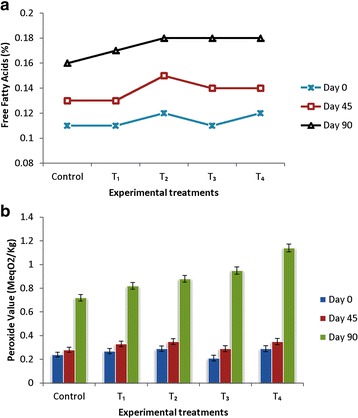
Oxidative Stability of Margarine Samples (**a**: Free fatty acids, %; **b**: Peroxide value, MeqO_2_/Kg)

Results of oxidative stability of margarine supplemented with chia oil have been presented in Fig. 3. Free fatty acids and peroxide values were used as indicators of oxidative stability. In current investigation, free fatty acids content of crude chia oil was 0.14% (oleic acid), free fatty acids content of margarine supplemented with 12% chia oil was 0.12%, which is within the allowable limits of 0.20%. Lower free fatty acids of chia oil offers better adaptation for the margarine industry. Free fatty acids of all the experimental samples and control went on increasing during the storage period of 90 days. After 90 days of storage period, free fatty acids of all the experimental margarines were within the allowable limit (0.3% European Standard EC No: ES-PDO-0105-0327-06.09.2011). The rise in free fatty acids of margarines during the storage period may be attributed to the lipases and metal ion contamination. Earlier investigation has shown that after 12 weeks of storage at 5 °C, free fatty acids of margarine were less than 0.2% [26]. Price of crude oils is mainly based on the concentration of free fatty acids. The edible oil manufacturers treat free fatty acids as impurity. For a better shelf and flavour stability of the finished product, they must be removed/neutralized. Considerable efforts and investments are required to lower the concentration of free fatty acids in edible oils. Higher magnitude of free fatty acids can lead to accelerated breakdown of peroxides to secondary and tertiary oxidation products. Estimation of peroxide value is a good indication of oxidation status of fats and oils [54]. Peroxide value of fresh and 45 days stored margarines (stored at 5 °C) were not different from each other and control. After 90 days of storage period, highest peroxide value was noted in T_4_ (1.14 MeqO_2_/kg), which is much lower than the maximum limit (10MeqO2/kg; European Standard: EC No: ES-PDO-0105-0327-06.09.2011). Addition of chia oil in margarine not only improved the concentration of omega-3 fatty acids but also altered the lipid peroxidation in supplemented margarines [55]. After 60 days of storage, TBA value of all the treatments and control were not different from freshly prepared samples (Table 6). Storage stability of margarine supplemented with 200 ppm tocopherol and 200 ppm rosemary extract were superior to 120 ppm tertiary butylated hydroxyl quinone [56]. During the storage of oils and fats, lipid oxidation is one of the major causes of quality deterioration. In order to retard the oxidative breakdown or to extend the shelf life of many foods, addition of antioxidants is required [57]. Due to the perceived toxicity, absorption and accumulation in body tissues and carcinogenic properties, synthetic antioxidants have been limited in numerous countries [58]. Use of ascorbic acid, rosemary and tocopherol to retard lipid oxidation has been described [59]. Addition of natural antioxidants considerably inhibited the oxidation in margarine [60]. In current investigation, oxidative stability of margarine was enhanced through the phenolic compounds of chai oil, without any addition of antioxidants.

**Table 6:** Thiobarbituric acid value of chia oil supplemented margarine in short term and mid term refrigeration storage

Treatments	0 Day	60 Days	120 Days	180 Days
Control	0.22 ± 0.02^f^	0.25 ± 0.03^f^	0.51 ± 0.02^e^	0.68 ± 0.01^d^
T_1_	0.22 ± 0.02^f^	0.26 ± 0.04^f^	0.55 ± 0.01^e^	0.73 ± 0.03^d^
T_2_	0.22 ± 0.02^f^	0.25 ± 0.01^f^	0.69 ± 0.03^d^	0.89 ± 0.04^b^
T_3_	0.22 ± 0.02^f^	0.26 ± 0.02^f^	0.82 ± 0.02^c^	0.94 ± 0.01^b^
T_4_	0.22 ± 0.02^f^	0.26 ± 0.05^f^	0.98 ± 0.06^b^	1.15 ± 0.05^a^

Values represent the mean ± standard deviation; *n* = 3

Means within the rows and columns with different superscript letters were significantly different (*p* < 0.05)

Control: Margarine Sample without Chia Oil

T_1_: Margarine Supplemented with 5% Chia Oil

T_2_: Margarine Supplemented with 10% Chia Oil

T_3_: Margarine Supplemented with 15% Chia Oil

T_4_: Margarine Supplemented with 20% Chia Oil

#### Sensory evaluation of margarine

**Fig. 4:**
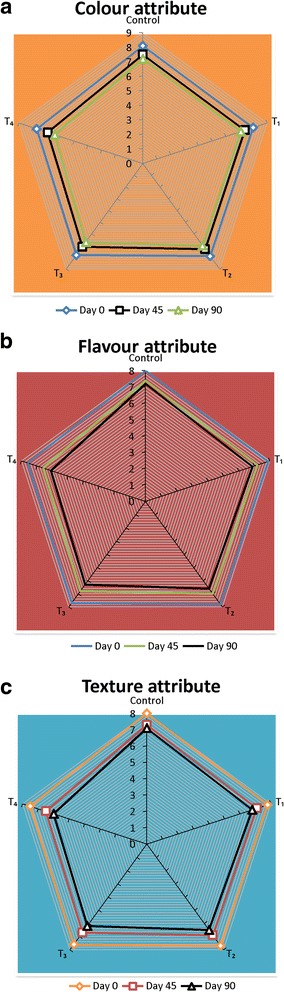
Sensory Characteristics of Margarine Samples (**a**: Colour attribute; **b**: Flavour attribute; **c**: Texture attribute)

Results of sensory evaluation of margarines supplemented with chia oil are presented in Fig. 4. At zero day, color, flavor and texture of all the treatments and control were not different from each other (*p* > 0.05). Storage period up to 45 days was non-significant for all the treatments and control. Color, flavor and texture score of 90 days stored T_4_ was less than other treatments and control (*p* < 0.05). After 45 days of storage period, decline in flavor score of T_4_ may be connected to the generation of peroxides that lead to the formation of odoriferous compounds, such as, aldehydes, ketones and alcohols. Nadeem et al. [61] recorded a strong correlation between peroxide value and flavor score. Sensory characteristics of a traditional sweet, prepared by mixing concentrated milk with margarine were better than sweet samples prepared from vanaspati. Omega-3 fatty acids of margarine were enhanced by fish oil, out of 195 analysts, only two perceived the fishy taste [62]. Lumor et al. [63] prepared *trans* free margarine from palm mid fraction and canola oil and sensory characteristics of *trans* free margarine were not different from the reference margarine.

## Conclusions

Chia oil at all levels enhanced the concentration of beneficial omega-3 & omega-6 fatty acids antioxidant characteristics of margarine. Supplemented margarines revealed low degree of changes in fatty acid profile, yielded the lower concentration of primary and secondary oxidation products. Sensory characteristics of margarine supplemented with 15% chia oil were not different from the control. Overall, omega fatty acids and antioxidant characteristics of *trans* free margarine can be enhanced by chia oil supplementation.
